# Same but different: aquatic prey capture in paedomorphic and metamorphic Alpine newts

**DOI:** 10.1186/s40851-019-0140-4

**Published:** 2019-07-26

**Authors:** Egon Heiss, Julia Grell

**Affiliations:** Institute of Zoology and Evolutionary Research, Friedrich-Schiller-University of Jena, Erbertstr. 1, 07743 Jena, Germany

## Abstract

Paedomorphosis describes the retention of larval characters in adult stages and is widespread amongst salamanders. Salamandrid newts exhibit facultative paedomorphosis, where paedomorphic and metamorphic adult forms coexist in the same population. Previous studies have shown that prey capture kinematics do not differ between paedomorphic and metamorphosed ambystomatid salamanders, despite diverging morphology and prey capture performance. It remained unclear, however, whether the stereotypy of prey capture kinematics across morphotypes is restricted to ambystomatids, or can be found in other salamander groups too. Here, we performed biplanar high-speed-recordings of the prey capture behavior in paedomorphic and metamorphic salamandrid newts and only found minor kinematic differences across morphotypes, suggesting that stereotypy across morphotypes is a more general feature within salamanders. We then compared anatomy of skull and hyobranchial skeleton, along with the physiological cross sectional area (PCSA) of the rectus cervicis muscle, the main muscle empowering suction feeding. Besides the overall morphological differences of the feeding apparatus, the PCSA of the rectus cervicis also differs significantly between morphotypes, being twice as large in paedomorphs. Accordingly, paedomorphs can exert more powerful suction strikes, which in turn may be one of the key factors why paedomorphs are more efficient in capturing elusive prey compared to metamorphs.

## Introduction

Paedomorphosis is defined as the retention of juvenile characters in sexually mature adults and is common in many salamander groups. In paedomorphic salamanders, adults forego metamorphosis and retain larval characters, such as the larval skull, hyobranchial musculoskeletal system, external gills, gill slits and fin folds [22, 32, 43, 56, 57, 66]. Paedomorphosis evolved multiple times independently in salamanders and larval development can be arrested in different ontogenetic stages [22, 74]. Next to obligate paedomorphosis where metamorphosis never occurs, some salamander groups have evolved facultative paedomorphosis, where both metamorphic and paedomorphic adults coexist in the same population [14, 23, 24, 63, 72]. Facultative paedomorphosis results in two heterochronic morphotypes with diverging morphologies of their feeding apparatus along with different prey capture performances [43, 58]. Aquatic salamanders use suction feeding where rapid oropharyngeal volume expansion creates a pressure drop in the oropharyngeal cavity that drives prey and surrounding water to flow into the gaping mouth [42]. Suction feeding relies on two fundamentally different flow regimes: unidirectional and bidirectional flow. Unidirectional flow is found in larval salamanders, where the engulfed water from the suction strike is released through the gill openings in the posterior oropharynx [43]. In contrast, bidirectional flow is typically found in metamorphosed salamanders that have sealed their gill openings during metamorphosis. In such bidirectional flow systems, the engulfed water is temporarily stored in the oropharyngeal cavity and then slowly released through the slightly opened mouth while prey is retained by the jaws [26, 43, 51]. Previous studies have shown that unidirectional flow systems are more efficient than bidirectional systems in capturing elusive prey [43, 58, 73], and larval salamanders create up to three times higher intraoral pressure drops compared to metamorphs of the same species and of comparable size [38]. Surprisingly, although larval salamanders create higher intraoral pressure drops and are more efficient in capturing elusive prey, their kinematics and motor pattern show no significant difference to metamorphosed salamanders [40, 64]. It has been suggested that the main factors leading to higher intraoral pressure drop and higher feeding efficiency in larval salamanders are based on the different flow regimes in larval and metamorphosed morphologies. However, direct comparison of feeding mechanics between larval and metamorphosed salamanders within the same species is only available for ambystomatids. In fact, it is unclear whether the lack in kinematic differences of feeding kinematics between larval and metamorphosed individuals is a general feature in salamanders, or only due to the potentially stereotypical nature of the feeding strike in ambystomatids [39].

Given that (i) paedomorphosis has evolved several times independently [74], different prey capture behavior across morphs could be present in other salamander groups and (ii) changes in muscle morphology, mass or physiology might, next to uni- vs. bidirectional flow systems, contribute to the diverging performance in larval and metamorphosed prey capture systems. Salamandrid newts are promising candidates to study the functional impact of paedomorphosis on feeding mechanisms, given the numerous cases of facultative paedomorphosis that provide the opportunity to study paedomorphs and metamorphs in adult stages of similar sizes from the same species.

Here, we compare prey capture kinematics in metamorphic and paedomorphic Alpine newts (*Ichthyosaura alpestris*) using biplanar high-speed recordings to test for kinematic differences across morphs. Based on the morphological differences of the prey capture system und diverging prey capture efficiency between paedomorphic and metamorphic newts [11], we hypothesize diverging prey-capture kinematics at least to some degree across the morphs. One of the main muscular systems empowering suction feeding in both larval and metamorphic salamanders is the rectus cervicis muscle (RC) that moves the hyobranchial system posteroventrally [42]. We test for differences in physiological cross-sectional area (PCSA; muscle volume divided by average fiber length) of the RC muscular system between metamorphs and paedomorphs. PCSA is a good performance indicator for muscles, as it is directly proportional to the maximal force that can be exerted by a muscle [5, 34]. Based on the anatomical differences between larval and metamorphosed salamanders [36, 42, 55], we expect larger RC with higher PCSA values in paedomorphic compared to metamorphic Alpine newts. If so, we would also expect higher flow rates in paedomorphs, resulting in higher peak velocities induced to the prey. Higher flow velocities induced by suction in paedomorphic Alpine newts could finally explain why they are more efficient in capturing elusive prey [11, 15].

## Materials & methods

### Newts

Five metamorphosed Alpine newts were collected in June 2011 in Lower Austria, Austria (private garden pond in the north of the city of Klosterneuburg) with collection permission (RU5-BE-18/022-2011) provided by the local government of Lower Austria. Additionally, four metamorphosed and four paedomorphic Alpine newts were collected between May and September 2012 in the Province of Bolzano (South Tyrol, see [24]), northern Italy with collection permission (63.01.05/120963), granted by the local government of the Province of Bolzano [24]. Identification of morphotypes (paedomorphs vs. metamorphs) and sexe (males vs. females) was in accordance to previous protocols [12]. For the experimental part of this study, only Alpine newts collected from Bolzano were used. For the morphological approach, metamorphs from Austria were included (Heiss et al., [29]). The animals were brought to the laboratory (Laboratory for Functional Morphology at the University of Antwerp, Belgium and Institute of Zoology and Evolutionary Research of the University of Jena, Germany) for high-speed recordings and morphological analyses. Animals were reared in aquaria with a 12-h dark/12-h light photoperiod at room temperature (ranging from 18 to 20 °C). Feeding was maintained twice a week with Chironomids, maggots (*Lucilia* sp.), and short winged crickets (*Gryllodes sigillatus*). The paedomorphic individuals were additionally fed *Daphnia*. Animal husbandry and experiments were approved by the Ethical Commission for Animal Experiments of the University of Antwerp (code: 2010–36) and the Committee for Animal Research of the State of Thuringia, Germany (animal experiment code: 02–008/15, animal husbandry code: J-SHK-2684-05-04-05-07/14).

### High-speed recordings and kinematic analyses

To record feeding kinematics, *I. alpestris* were habituated to feed in a small glass aquarium (base area: 12 × 30 cm, height: 20 cm) where they were filmed at room temperature with two digital high-speed cameras (Photron FASTCAM model 100KC, Photron limited, Germany) in lateral and ventral views at a frame rate of 500 Hz. The newts were offered a living *Chironomus* larva with an average length of 10.4 ± 0.8 mm (mean ± s.d.; *n* = 30) at a time in front of the head. To avoid distortive effects of different prey types on the prey-capture behaviour (Maglia and Pyles, [8]; Deban [47]), living *Chironomus* larvae were used as standardized prey items. *Chironomus* larvae were also used because they are a natural prey [13, 33] and all newts readily fed on them.

The camera for the lateral recordings was equipped with a 60 mm macro lens, whereas the camera for the ventral view was equipped with a standard 50 mm lens. During recordings, illumination was provided by spotlights with reduced heat emission (model VD-7000 LP, Vision Devices GmbH, Germany). As reference, two rulers were attached to the aquarium, perpendicular to the camera views.

**Fig. 1:**
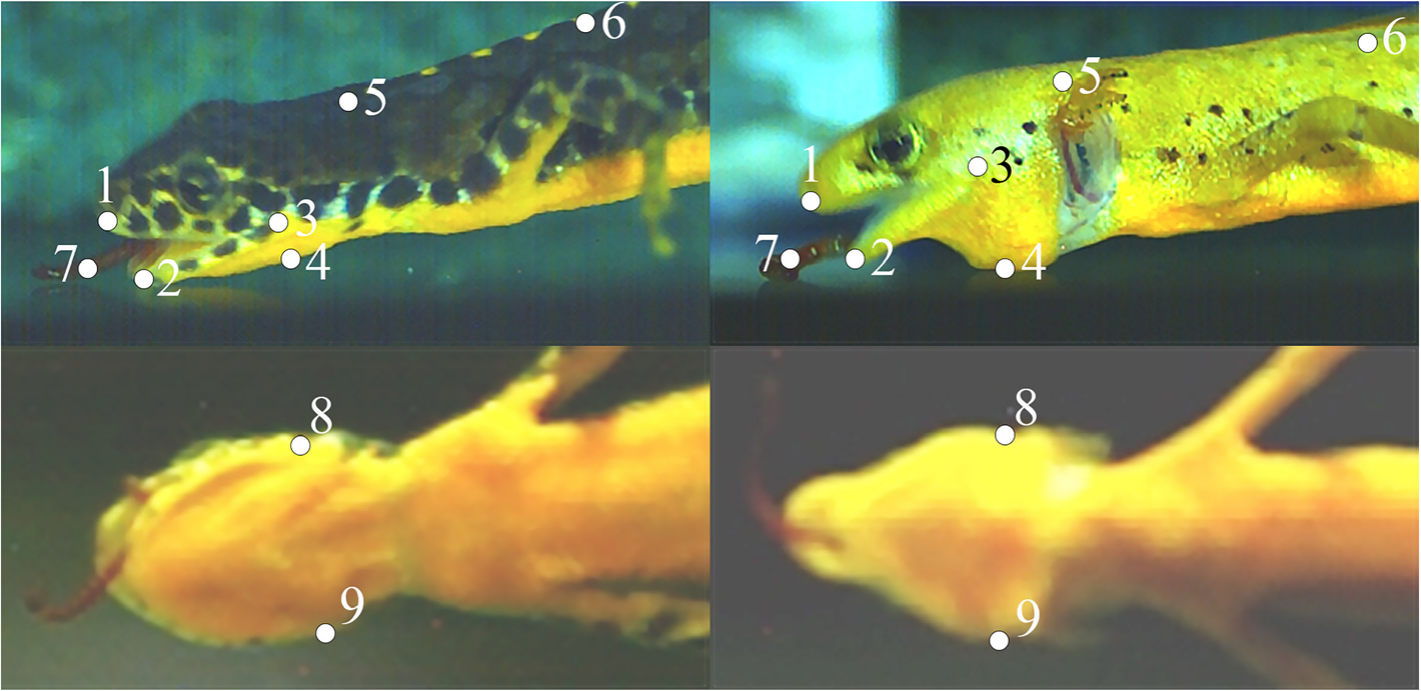
Frame-shots showing the landmarks used to calculate movements and kinematic variables in metamorphs (left) and paedomorphs (right) in lateral (top) and ventral (bottom) views: 1, upper jaw tip; 2, lower jaw tip; 3, jaw joint; 4, hyobranchium; 5, nape; 6, trunk reference; 7, estimated center of mass of prey; 8, left jaw joint; 9, right jaw joint

Four metamorphosed (two males and two females with snout-vent-lengths of 44.2 ± 3.2 mm; mean ± s.d.) and two paedomorphic (both females with snout–vent lengths of 43 and 45 mm, respectively) Alpine newts were recorded at the University of Jena, as described above. From all recordings, eight videos (eight lateral with synchronized ventral videos) per individual were chosen for further analyses based on quality criteria. Additionally, six high-speed recordings of a third paedomorphic animal (female, lateral view only; snout-vent-length: 43.5 mm) that was recorded in 2012 at the University of Antwerp (using a Redlake Motion-Pro HR1000a; Redlake Digital Imaging Systems, IDT Vision, USA) was available for this study, resulting in a total of 54 feeding events ready for analyses. The horizontal (x-axis) and vertical (y-axis) coordinates of previously defined landmarks (Fig. 1) were tracked frame-by-frame using the Simi Motion software package (SIMI Reality Motion Systems, Germany). Landmarks were based on those used by other studies on salamander prey capture ([Deban, [8–10, 26], Reilly, [60], Reilly, [61, 65]) to allow direct comparison of kinematics.

From the 2D displacement of the landmarks, the following movements were calculated: (i) from lateral recordings: jaw movement (distance between the tips of the upper and lower jaw), head rotation (dorsoventral angle displacement of the head relative to the trunk), hyobranchial depression (distance between the jaw joint and throat where maximum depression occurs) and velocity of the prey item (2D movement of the estimated centre of mass of the prey) and (ii) from ventral recordings: lateral head expansion (2D displacement measured between the jaw joints).

From these movements, 11 kinematic variables were extracted in analogy with previous research on prey-capture biomechanics in salamanders ([8, 60, 61, 65]): 1) maximum gape (maximum distance between the upper and lower jaw tip); 2) mouth opening duration (time from mouth opening until maximum gape); 3) mouth closing duration (time from maximum gape until end of mouth closing); 4) total gape cycle duration (time from mouth opening until end of mouth closing); 5) maximum hyobranchial depression (maximum distance between jaw joint and hyobranchium); 6) time to start hyobranchial depression (time from start of the gape cycle until first posteroventral rotation of the hyobranchium); 7) duration of hyobranchial depression (time from start of hyobranchial depression until maximum depression); 8) maximum head elevation (maximum head lift in degree in sagittal plane); 9) duration of head elevation (time from start of the gape cycle until maximum head lifting); 10) maximum lateral expansion (maximum displacement between left and right jaw joints); 11) maximal prey velocity (maximal velocity induced to the prey item from mouth opening till end of ingestion). Lateral expansion was only measured for two out of the three paedomorphic *I. alpestris* (see above).

### Micro-computed tomography (μCT)

Five metamorphic (collected in Lower Austria, see above) and two paedomorphic specimens were used for μCT-analysis. While data for metamorphosed *I. alpestris* (all males with snout-vent-lengths of 42.6 ± 2.9 mm; mean ± s.d.) are used from a previous study [25, 28], data from paedomorphs (both females with snout–vent lengths of 45 and 43.5 mm, respectively) were newly gained. One paedomorphic animal also provided the additional lateral-view-only high-speed-recordings (see above), while the other animal was used for morphological analyses only. All animals were euthanized and prepared as described in Heiss [24] and Heiss et al. [25]. In short, specimens were euthanized by immersing them in an aqueous solution of 0.05–0.5% MS222 buffered to pH 7.2 [44], fixed in 4% formaldehyde, dehydrated in a graded ethanol series, contrasted in a solution of 1% elemental iodine in absolute ethanol, rinsed and mounted in Falcon tubes. Scans of the whole heads were acquired using a SkyScan 1174 (Bruker, Belgium) μCT scanner with a source voltage of 50 kV and an isovolumetric voxel resolution of 7.39 μm. After μCT scans were performed, the newts were dissected to estimate the physiological cross-sectional area of the rectus cervicis muscle (see below). The vouchers of both paedomorphic newts are kept in the State Museum of Natural History Stuttgart (SMNS 16344 and SMNS 16345).

### 3D reconstruction

After image acquisition, image stacks were imported into the 3D software package AMIRA 4 (FEI Visualization Sciences Group, Merignac Cedex, France). Based on tomographic image data, relevant structures were segmented either manually or by threshold segmentation and visualized via surface renderings. Volumes of the manually segmented rectus cervicis muscles were measured via Amira Material Statistics tool. The rectus cervicis is an extension of the rectus abdominis muscle of the ventral trunk musculature and given its ‘blurry’ and not always detectable origin (i.e., the first tendinous inscription) in μCT scans, the anterior margin of the pericardium was defined as the posterior margin of the ‘functional rectus cervicis’. This is justified by the fact that, according to previously published studies [17, 20, 21, 55], the first tendinous inscription is located close to the anterior margin of the pericardium.

### Determining fiber length and PCSA

Five metamorphic and two paedomorphic Alpine newts (same individuals as used for μCT scanning) were used for fiber-length measurements. To measure the mean muscle fiber length, the individuals were dissected and prepared as described in Heiss et al. [25]. In short, the rectus cervicis muscles were carefully removed and immersed in 30% hydrous nitric acid solution for up to 24 h to dissolve the collagenous tissue surrounding the muscle fibers (Nauwelaerts et al., [53]). Next, muscles were rinsed in tap water and muscle fibers were carefully separated using two fine pins under a Carl Zeiss GSZ stereo microscope (Carl Zeiss, Jena, Germany) and covered with a coverslip. Digital micrographs were taken using an Olympus BX21 light microscope (Olympus, Japan), and the lengths of 20 randomly selected muscle fibers were measured using the open source software package Inkscape 0.92.4. The PCSA of rectus cervicis were calculated from muscle volume divided by mean fiber length [50]. Muscle volume was measured from the μCT scans, as described above.

### Statistics

Kinematic variables were tested for normal distribution and homogeneity and as the data violated the assumptions for parametric statistics, nonparametric tests were performed. We used a series of 11 Mann-Whitney-U tests to determine the effect of morphotype (i.e. paedomorphs vs. metamorphs) on the prey-strike kinematics. In order to account for the multiple tests performed, the *p*-value was corrected after Bonferroni to *p* ≤ 0.0046.

Similarly, morphological variables did not fulfill the requirements for parametric tests, so three separate Mann-Whitney-U tests were performed to reveal differences between volume, fiber length and PCSA of the rectus cervicis muscle across morphotypes. Data from left and right muscles were pooled to obtain higher statistical power. To account for the three serial tests performed, the *p*-value was corrected after Bonferroni to *p* ≤ 0.017.

All statistical tests were performed using SPSS Statistics 25 (IBM, USA) software packages.

## Results

### Kinematics

**Fig. 2:**
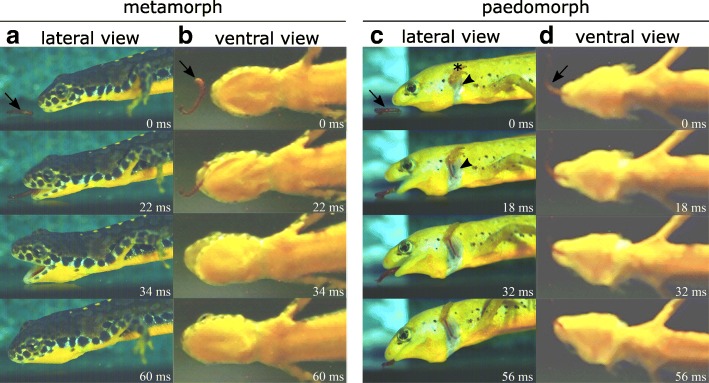
Frame-shots showing the prey strike in metamorphic (**a** and **b**) and paedomorphic (**c** and **d**) alpine newts from lateral (**a** and **c**) and ventral views (**b** and **d**). The prey (*Chironomus* larva) is indicated by arrows. Note that the paedomorphic animal exhibits well-visible gill openings (arrowhead) just beneath the external gills (asterisk), which are absent in the metamorphosed animal

**Fig. 3:**
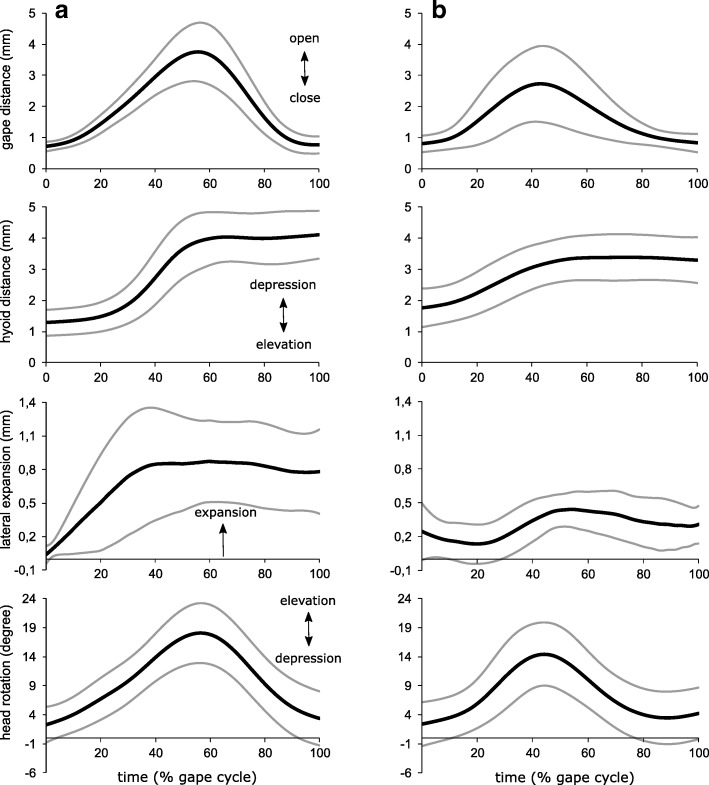
Kinematic profiles showing gape, hyobranchium (hyoid), lateral head, and vertical head movements in metamorphic (**a**) and paedomorphic (**b**) Alpine newts. Bold and black lines indicate the mean values, while the grey and slender lines are ± standard deviation. The X-axes are normalized as percentage of total gape cycle

From a qualitative perspective, the suction strike of metamorphs and paedomorphs are very similar and the following description refers to both forms if not noted otherwise: The prey strike is initiated with fast gape opening by dorsal head rotation and lower jaw depression, followed by hyobranchial depression and prey is accelerated into the gaping mouth (Figs. 2 and 3). Lateral expansion of the head during jaw opening was only evident in metamorphs. Immediately after the gape has reached its peak, it started closing by head depression and lower jaw elevation to entrap the prey, while the hyobranchium remained depressed and lateral head expansion remained stationary in metamorphs.

**Fig. 4:**
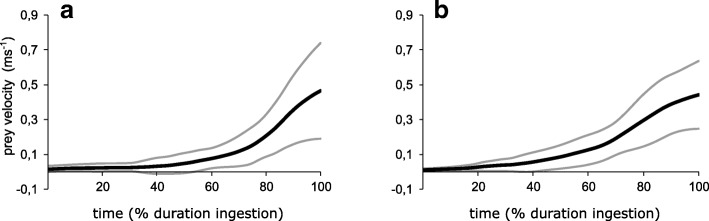
Velocity of prey movements towards the Alpine newt’s mouth, induced by suction (**a** metamorphs; **b** paedomorphs). The X-axes are normalized to the duration of prey ingestion, i.e. the time from the first prey movement towards the newt until prey disappeared in the mouth

From a quantitative perspective, subtler differences could be detected between morphs. The series of Mann-Whitney-U tests (Table 1) revealed significant differences in the duration of gape opening (*p*<0.001; U = 98.5) and gape closing (*p* = 0.003; U = 187) but not between the duration of total gape cycle (*p* = 0.344; U = 298.5). The maximum gape distance (*p* = 0.006; U = 197) was not significantly different between morphs after Bonferroni-correction. Similarly, the delay of hyobranchial depression relative to the start of gape opening (*p* = 0.015; U = 230.5) and the duration of hyobranchial depression (*p* = 0.23; U = 287) showed no significant difference. In contrast, the maximum distance the hyobranchium was depressed was significantly greater in metamorphs compared to paedomorphs (*p*<0.001; U = 29). The duration of head elevation (*p* = 0.006; U = 198.5) and the maximum degree of head elevation (*p* = 0.193; U =278) showed no significant difference between the two morphs. Lateral expansion of the head (*p*<0.001; U = 50) was significantly higher in metamorphs. The maximum velocity (*p* = 0.93; U = 347) induced to the prey did not differ between the two morphs (Fig. 4).

**Table 1:** Kinematic variables and series of Mann-Whitney-U tests comparing paedomorphic and metamorphic Alpine newts. Significance level was adjusted to *p* ≤ 0.0046 after simultaneous Bonferroni correction

Variable	Paedomorphs	Metamorphs	Mann-Whitney-U	*P*-Value
Duration gape opening (ms)	22 ± 5.55	30.89 ± 6.44	98.5	<0.001
Duration gape closing (ms)	31.09 ± 7.35	25.5 ± 3.21	187	0.003
Duration gape cycle (ms)	53.09 ± 10.51	56.19 ± 7.15	298.5	0.344
Maximum gape (mm)	2.94 ± 1.3	3.87 ± 0.93	197	0.006
Time to start hyobranchial depression (ms)	1 ± 2.02	4.56 ± 6.41	230.5	0.015
Duration hyobranchial depression (ms)	38 ± 9.82	41.6 ± 10.61	284	0.23
Maximum hyobranchial depression (mm)	1.85 ± 0.47	3.01 ± 0.56	29	<0.001
Duration head elevation (ms)	27.82 ± 9.44	31.81 ± 4.75	198.5	0.006
Maximum head elevation (deg)	16.58 ± 4.84	18.6 ± 4.8	278	0.193
Lateral head expansion (mm)	0.62 ± 0.16	1.18 ± 0.39	50	<0.001
Maximum prey velocity (ms^−1^)	0.47 ± 0.18	0.49 ± 0.25	347	0.93

### Morphology

The morphology of the skull and hyobranchial apparatus of metamorphosed and paedomorphic salamandrids is described in detail elsewhere [16–18, 20, 21, 32, 55, 62]; we focus mainly on the overall head morphology of both morphs and center on the hyobranchium and the rectus cervicis muscle system. Description and terminology largely follows Drüner [17, 18], Francis [21], Özeti and Wake [55], Findeis and Bemis [20].

**Fig. 5:**
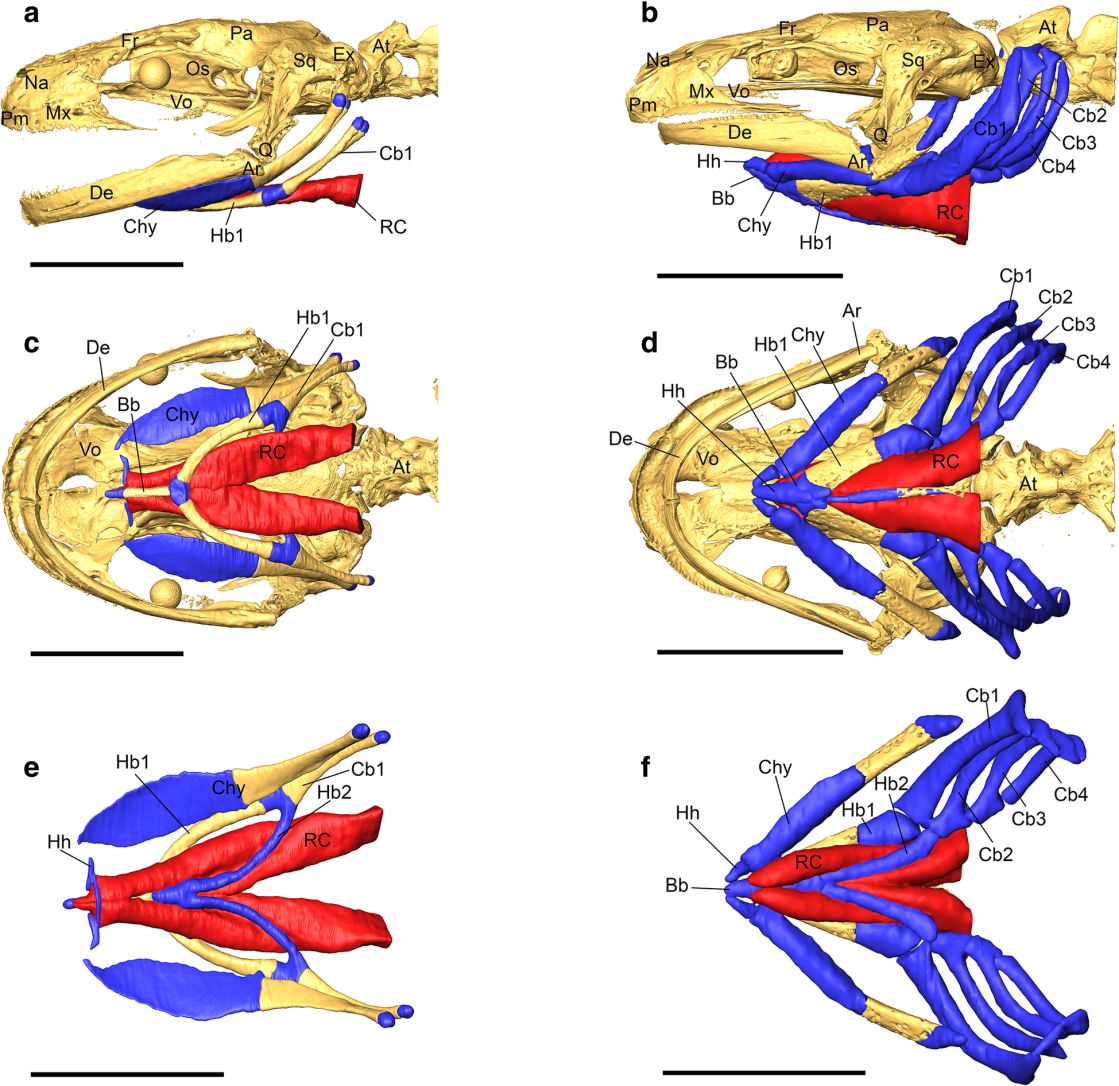
Morphology of skull, hyobranchial skeleton and rectus cervicis musculature in metamorphic (**a**, **c** and **e**) and paedomorphic (**b**, **d** and **f**) Alpine newts in lateral (**a** and **b**), ventral (**c** and **d**) and dorsal views (**e** and **f**). The skull is virtually removed in **e** and **f** to allow free view to the dorsal face of the hyobranchial skeleton and the rectus cervicis musculature. Golden colour indicates bony tissue, blue cartilage and the rectus cervicis muscle is indicated in red. Abbreviations: Ar, articular; At, atlas (cervical vertebra); Bb, basibranchial; Cb1–4, ceratobranchial 1–4; Chy, ceratohyal; De, dentary; Ex, exoccipital; Fr, frontal; Hb1–2, hyobranchial 1–2; Hh, hypohyal (=radial); Mx, maxillary; Na, nasal; Os, orbitosphenoid; Pa, parietal, Pm, premaxillary; Q, quadrate; RC, rectus cervicis musculature; Sq, squamosal; Vo, vomerine. Scale bars: 5 mm

Overall, the horizontal skull shape is roundish in metamorphs, but tapered in paedomorphs (Fig. 5). In both morphs, the anterior skull elements that build up the dorsal jaw system (i.e. premaxillary and maxillary) are well developed. The suspension of the lower jaw, i.e., squamosal and quadrate, are robust elements in both forms (Fig. 5). The lower jaw consists of articular and dentary, and is relatively gracile in metamorphs but robust in paedomorphs (Fig. 5). The dorsal plates of the skull, i.e. nasal, frontal, and parietal are well-developed in both forms and so is the occipital region that bears the occipital condyls that articulate with the cervical vertebra (Fig. 5). From ventral view, the parasphenoid covers most part of the roof of mouth and is similarly shaped in both morphs. More subtle differences are evident from the shape and position of the vomers. They are broad anteriorly and bear an elongated posterior process in metamorphs, while they are restricted to the anterior roof of mouth in paedomorphs.

More prominent differences concern the hyobranchium. In both morphs, the hyobranchial system lies between the mandibular rami in the floor of mouth but is significantly larger and consists of more elements in paedomorphs (Fig. 4). In both morphs, the hyobranchial skeleton consists of a central unpaired element, the basibranchial, that is bony in metamorphs and cartilaginous in paedomorphs (Fig. 5). The anterior tip of the basibranchial articulates with the hypohyals, small cartilaginous elements that articulate with the ceratohyals in paedomorphs but have lost this connection in metamorphs (Fig. 5). In metamorphs, left and right hypohyals (also referred to as “radii”), are connected by a cartilaginous dorsal arch. This arch is absent in paedomorphs. The posterior tip of the basibranchial articulates with hypobranchial 1 and hypobranchial 2 in both morphs. While hypobranchial 2 is cartilaginous in both forms, hypobranchial 1 is entirely bony in metamorphs but only the anterior two-thirds are bony in paedomorphs. On their posterior tip, hypobranchial 1 and 2 articulate with the ceratobranchial 1 in both morphs. Additionally, paedomorphs bear the ceratobranchials 2, 3 and 4 which are absent in metamorphs (Fig. 5).

The rectus cervicis muscle (Fig. 5a-f) is a direct extension of the rectus abdominis (ventral trunk musculature), originates on the first tendinous inscription and consists of two portions: the rectus cervicis superficialis and the rectus cervicis profundus. The rectus cervicis superficialis attaches on the posteriormost portion of the basibranchial in both morphs. The deeper portion, the rectus cervicis profundus, attaches on the anterior portion of the basibranchial (both morphs), plus the cartilaginous arch that connects left and right radius in metamorphs (this arch is absent in paedomorphs, see above). In this study, superficialis and profundus portions of the rectus cervicis muscle are treated as one functional entity that is simply referred to as “rectus cervicis”. The rectus cervicis is larger in paedomorphs, as shown by μCT imaging (Fig. 5) and subsequent calculations (see Table 2). In detail, the rectus cervicis in metamorphs bears a volume (measured from insertion to first tendinous inscription) of 2.71 ± 0.98 mm^3^ (mean ± s.d.), while its volume is nearly twice as large in paedomorphs (5.8 ± 0.22 mm^3^). In contrast, average rectus cervicis fiber length in metamorphs (5.55 ± 0.26 mm) does not differ from the fiber length in paedomorphs (5.45 ± 0.95 mm). Diverging volume but similar fiber size lengths results in different rectus cervicis PCSA in metamorphs (0.49 ± 0.17 mm^2^) and paedomorphs (1.08 ± 0.15 mm^2^).

**Table 2:** Volume, fiber length and PCSA of the rectus cervicis muscle in paedomorphic and metamophic *I. alpestris*. Values are mean ± 1 standard deviation. The Mann-Whitney-U tests compare values between the two morphs. Significance level was adjusted to *p* ≤ 0.017 after simultaneous Bonferroni correction.

	Metamorph (*n* = 10)	Paedomorph (*n* = 4)	Mann-Whitney-U	*P*-value
RC volume (mm^3^)	2.71 ± 0.98	5.8 ± 0.22	0	0.005
RC fiber length (mm)	5.55 ± 0.26	5.45 ± 0.95	20	1
RC PCSA (mm^2^)	0.49 ± 0.17	1.08 ± 0.15	0	0.005

## Discussion

Despite major morphological differences of the feeding system, only minor differences in prey capture kinematics were detected between paedomorphic and metamorphic Alpine newts. Only duration of gape opening and gape closing, along with maximum hyobranchial depression and lateral head expansion, showed significant differences across morphotypes. The most remarkable of those differences is associated with lateral head expansion: In the present study we found lateral head expansion in metamorphs, but not in paedomorphs. Previous studies have hypothesized that lateral head expansion is achieved in salamanders by abduction of the suspension (i.e. squamosal) of the lower jaw system relative to the skull [30, 31, 42, 52]. We only found negligible lateral head expansion in paedomorphs but a clear signal in metamorphs. Does this necessarily mean that metamorphs do abduct their squamosal relative to the skull and paedomorphs do not? We performed light film recordings and measured the expansion in the region of the jaw joints but cannot exclude that the lateral expansion measured was based on abduction of the suspension of the lower jaw (pleurokinesis sensu [31]), lateral hyobranchial movement or due to the passive expansion of the gular region caused by the inflowing water. Only biplanar X-ray approaches with implanted radiopaque markers in the squamosal and the skull will be able to answer the question whether squamosal abduction as form of cranial pleurokinesis is present in salamanders.

The minor differences in feeding kinematics between paedomorphic and metamorphosed Alpine newts corroborate the results from previous studies that neither feeding kinematics nor motor patterns change across metamorphosis in salamanders [40, 64]. Accordingly, the present study on salamandrid newts, along with the findings of Lauder and Shaffer [40] and Shaffer and Lauder [64] on ambystomatid salamanders suggest that the conservation of movement and motor patterns across metamorphosis is a general feature in salamanders. But why should a movement pattern be conserved across metamorphosis where a fundamental reorganization of the entire craniovisceral musculoskeletal system occurs? It might be argued that the general requirements for suction feeding are similar for vertebrates, regardless of their morphology or phylogenetic position. In fact, rapid oropharyngeal volume expansion is common in all suction-feeding vertebrates, as described in cartilaginous and bony fishes [1, 3, 4, 41, 68, 75], lissamphibians [6, 54, 59, 65], turtles [37, 46, 67], mammals [35, 48, 70, 71] and birds [19]. In most cases, oropharyngeal volume expansion is achieved by rapid gape opening and hyobranchial/hyolingual depression, resulting in similar kinematics. Based on the morphological constraints of vertebrates, there are few functional alternatives left to create suction and the movement patterns are similar, even if they have evolved convergently in many cases (but see [7] for an exceptionally different suction strike mechanism). Accordingly, the similar movement pattern of the suction strike in paedomorphic and metamorphic Alpine newts is most probably the result of the physical demands imposed by the dense and viscous medium water, along with the morphological constraints to create fast oropharyngeal volume expansion.

The different flow regimes in uni- vs. bidirectional suction feeders seem to have little effect on the first half of the suction strike, but during the second half of the strike, the flow regime does impact the suction mechanism [43]. Specifically, after the negative pressure peak induced by oropharyngeal volume expansion in the first half of the suction strike, the momentum of the inflowing water gradually increases the oropharyngeal pressure towards supra-ambient levels in the second half of the strike [26, 43]. If the inflowing water cannot be released through gill openings, the duration of the negative oropharyngeal pressure phase is shorter and its amplitude smaller, while the phase of increased oropharyngeal pressure lasts longer and the positive pressure peak is higher compared to unidirectional systems [43]. Accordingly, the energy imparted to the water by a salamander during a suction strike is higher in unidirectional, i.e., larval, morphologies [43]. However, bidirectional flow systems are not inefficient at all. To minimize or delay the oropharyngeal pressure increase in the second half of the suction strike, bidirectional suction feeders actively or passively expand their pharyngeal or esophageal cavity to temporarily store the inflowing water volume [2, 26, 46]. To directly compare uni- and bidirectional suction feeding systems, Lauder and Reilly [38] performed experiments in which intraoral pressure during suction feeding in the axolotl (*Ambystoma mexicanum*) was measured first in its natural state, and then after the gill openings were sutured close. With other words, a unidirectional suction feeder was transformed to a bidirectional suction feeder with everything else being equal. These experiments revealed that the magnitude of the pressure drop in the early stages of the suction strike did not differ between pre- and post-treatments, while the magnitude and duration of the pressure rise in the later stages of the strike differed significantly. Interestingly, even if the axolotls’ gill slits were sutured close, their intraoral pressure drop was still higher than in naturally metamorphosed ambystomatids with comparable body size. So, which factors influence suction feeding performance in salamanders across metamorphosis next to the flow regime?

Metamorphosis in salamanders results in dramatic changes in the craniovisceral musculoskeletal system, affecting suction feeding performance and prey preferences. For example, the skull in pre-metamorphic salamanders is more tapered, the hyobranchial skeleton is larger and bears more posterior elements and it was hypothesized that some muscles decrease in mass during metamorphosis [43]. Indeed, our study showed that the rectus cervicis muscle that pushes the hyobranchial apparatus posteroventrally and is therefore one of central muscles empowering suction feeding, is significantly larger with a PCSA that is roughly twice as large in paedomorphic Alpine newts compared to metamorphs. Given that the PCSA is directly proportional to the maximal force a muscle can exert, paedomorphs can depress their larger hyobranchial skeleton with higher forces and therefore move a larger volume of water in a given time. If the head is tapered and the gape opening smaller and restricted anteriorly, this may result in higher suction force and more precise suction strikes. Accordingly, next to the unidirectional flow regime that delays intraoral pressure increase, the larger hyobranchial system, along with a larger and stronger RC, may explain why the suction strike with a larval morphology can impart more energy to the water. Still, our results show that the velocity of the prey induced by suction does not differ between paedomorphic and metamorphic Alpine newts. However, we only tested the effect on one prey type lying on the ground; the capability in paedomorphs for more forceful suction strikes could come into play when capturing more elusive or larger prey. In fact, Denoel [11] showed that paedomorphic Alpine newts were more successful in capturing three out of four elusive prey types [11]. Next to different capabilities for capturing elusive prey, also different prey preferences were found between morphs [11, 15]. Furthermore, it has been shown that paedomorphic and metamorphic Alpine newts from the same population partition their trophic habitats [14, 45]. Depending on size and heterogeneity of habitats, paedomorphs tend to live in the entire water column and deep benthic areas where they mainly capture small elusive prey, such as *Daphnia* and other small pelagic invertebrates while metamorphs live close to the shoreline and mainly rely on terrestrial arthropods that fell into the water [14].

Why do paedomorphic Alpine newts restrain from capturing terrestrial arthropods that fell into the water and instead mainly capture smaller prey, including plankton? It has been suggested that the main reason is associated with smaller gapes and the presence of labial lobes that further restrict the gape opening in paedomorphs [11, 64] but first, also metamorphosed newts bear labial lobes in their aquatic stage [49, 69] and second, our study found no significant difference in maximal gape opening between morphs (after Bonferroni-correction). So gape alone is improbable as an explanation of why paedomorphic Alpine newts avoid larger prey and other factors should be considered. Specifically, next to prey capture, intraoral prey manipulation is an integral—though often overlooked—stage of the feeding process. A recent study showed that metamorphosed newts process prey by cyclically rasping it against the vomerine dentition by loop motions of the tongue [27]. However, during metamorphosis, the vomerine dentition pattern and hyobranchial musculoskeletal system (i.e., tongue) undergoes drastic changes. Without such major changes in the processing apparatus (and no movable tongue), newts with larval morphologies cannot employ the same processing mechanism observed in metamorphs. For now, little is known on processing mechanisms employed in larval salamanders but diverging processing techniques might be considered an important factor that leads to different competences to handle prey, ultimately resulting in different prey preference and partition of trophic habitats between metamorphic and paedomorphic Alpine newts. Partition of trophic habitats finally decreases intraspecific competition in adults and favors the maintenance of facultative paedomorphism in populations [11].

## Conclusions

Despite the remarkable differences in head morphology that result in different flow regimes, paedomorphic and metamorphic Alpine newts only show minor differences in suction feeding kinematics. Nonetheless, the larger rectus cervicis muscle with larger PCSA, along with a larger hyobranchial skeleton in paedomorphs results in higher suction force potential, which may be one of the key factors why paedomorphs are more efficient in capturing elusive aquatic prey than metamorphs. The present study implies that (i) stereotypy of aquatic prey capture kinematics is a more general rule across larval and metamorphic morphologies in salamanders and (ii) despite similar kinematics, paedomorphs have the potential to produce higher suction forces during aquatic prey strikes and probably use different prey processing mechanisms than metamorphs. Diverging suction feeding efficiencies and different prey processing mechanisms might have selective advantages by fostering the partitioning of trophic habitats between morphs and ultimately favoring the maintenance of facultative paedomorphism in salamanders.

## Data Availability

The datasets during and/or analysed during the current study available from the corresponding author on reasonable request.

## Abbreviations

Ar: Articular
At: Atlas (cervical vertebra)
Bb: Basibranchial
Cb1–4: Ceratobranchial 1–4
Chy: Ceratohyal
De: Dentary
Ex: Exoccipital
Fr: Frontal
Hb1–2: Hyobranchial 1–2
Hh: Hypohyal (=radial)
Mx: Maxillary
Na: Nasal
Os: Orbitosphenoid
Pa: Parietal
PCSA: Physiological cross sectional area
Pm: Premaxillary
Q: Quadrate
RC: Rectus cervicis muscle
s.d.: Standard deviation
Sq: Squamosal
Vo: Vomerine
μCT: Micro computed tomography
